# MRI-guided thrombolysis for lenticulostriate artery stroke within 12 h of symptom onset

**DOI:** 10.1038/s41598-022-11459-3

**Published:** 2022-05-06

**Authors:** Jianying Zhang, Qingke Bai, Zhenguo Zhao, Yiting Mao, Qiang Dong, Wenjie Cao

**Affiliations:** 1 Department of Neurology, Shanghai Pudong New Area People’s Hospital, Shanghai, People’s Republic of China; 2 Department of Neurology, Huashan Hospital, Fudan University, No. 12 Wulumuqi Zhong Rd, Shanghai, 200040 People’s Republic of China; 3 Department of Radiology, Shanghai Pudong New Area People’s Hospital, Shanghai, People’s Republic of China; 4 State Key Laboratory of Medical Neurobiology, Fudan University, Shanghai, People’s Republic of China; 5 National Clinical Research Center for Aging and Medicine, Huashan Hospital, Fudan University, Shanghai, People’s Republic of China

**Keywords:** Computational biology and bioinformatics, Stroke

## Abstract

Stroke thrombolysis treatment is generally administered within 4.5 h, but a greater time window may be permitted depending upon the ischemic penumbra on neuroimaging. This observational cohort study investigated the outcomes of thrombolysis given within 12 h after symptom onset of lenticulostriate artery stroke. The population comprised 160 patients. Thrombolysis was administered via tissue plasminogen activator, alteplase (TPA). Thrombolysis was indicated by a mismatch between diffusion-weighted imaging (DWI) and T2-weighted imaging (T2WI), that is, an acute ischemic lesion on DWI without a corresponding lesion on T2WI. Demographics and medical history were compared with the modified Rankin scale (mRS) score, to reflect outcome. Patients with a favorable clinical outcome (mRS 0–1) had significantly lower hypertension, baseline NIH Stroke Scale (NIHSS) score, and admission systolic/diastolic blood pressure compared with patients with mRS 2–6. Lower admission systolic blood pressure and NIHSS score were significantly associated with favorable outcome. In patients either with IV-TPA within 4.5 h, or between 4.5 and 12 h, lower admission systolic blood pressure and/or NIHSS score similarly independently predict favorable outcome. However, in all groups, the onset-to-treatment time did not significantly influence the outcomes. We conclude that in our cohort higher admission systolic blood pressure and higher baseline NIHSS and not time were associated with poor outcome in patients with magnetic resonance-guided thrombolysis within 12 h of isolated lenticulostriate artery stroke, therefore loosening the traditionally perceived dependency of outcome on time.

## Introduction

Infarction of the lenticulostriate artery (LSA) is cerebral ischemia that involves territories supplied by the deep perforating branches of the middle cerebral artery. These include the periventricular area, basal ganglia, and internal capsule, which are primarily involved in the key neural fiber pathway. LSA infarction accounts for ~ 13.5% of cerebral infarctions that are related to anterior circulation^1,2^. Progressive motor deficits are common in LSA infarction and may cause severe disability^3^

For patients with acute ischemic stroke, intravenous tissue plasminogen activator (IV-TPA) is the only effective medical treatment known to improve outcomes^4,5^. Recently, high-resolution magnetic resonance angiography at 7 T has been used to detect the recanalization of LSAs in patients with acute LSA infarction^6^. The time window for administering IV-TPA to the patient with ischemic stroke may be extended, based on the ischemic penumbra on neuroimaging^7^. However, the outcomes of IV-TPA for patients with LSA stroke who present within an extended time window has not been addressed well, nor the predictive factors that may be associated with clinical outcomes.

Magnetic resonance imaging (MRI)-based selection trials showed that IV-TPA benefited patients with acute ischemic stroke in a setting of unknown onset time and a mismatch between diffusion-weighted imaging (DWI) and fluid-attenuated inversion recovery (FLAIR)^8,9^. It is unclear whether a mismatch between DWI and T2WI, which indicates a salvageable tissue window, is also suitable for administration of IV-TPA beyond the 4.5-h time window in patients with LSA stroke.

The present retrospective observational cohort study compared the outcomes of MRI-guided administration of IV-TPA within 4.5–12 h after LSA stroke symptom onset, to that of IV-TPA within the accepted time window of 4.5 h. Factors that may be associated with the clinical outcome were also explored.

## Methods

### Study population and study protocol

Initially, data for 1462 patients was collected. These patients had suspected stroke, satisfied the guidelines for administration of IV-TPA, and were treated from July 2007 to April 2018 at Pudong New Area People’s Hospital, a single hospital. Of the 1462 patients, 601 (41%; 601/1462) with MRI DWI/T2WI mismatches underwent thrombolysis within 12 h after the acute onset of symptoms. Among these, 160 (26.6%; 160/601) had a confirmed stroke affecting only the LSA territory. The remaining 861 (59%; 861/1462) patients had a DWI/T2WI match and were denied IV-TPA irrespective of the time window in which they presented to the hospital.

**Figure 1:**
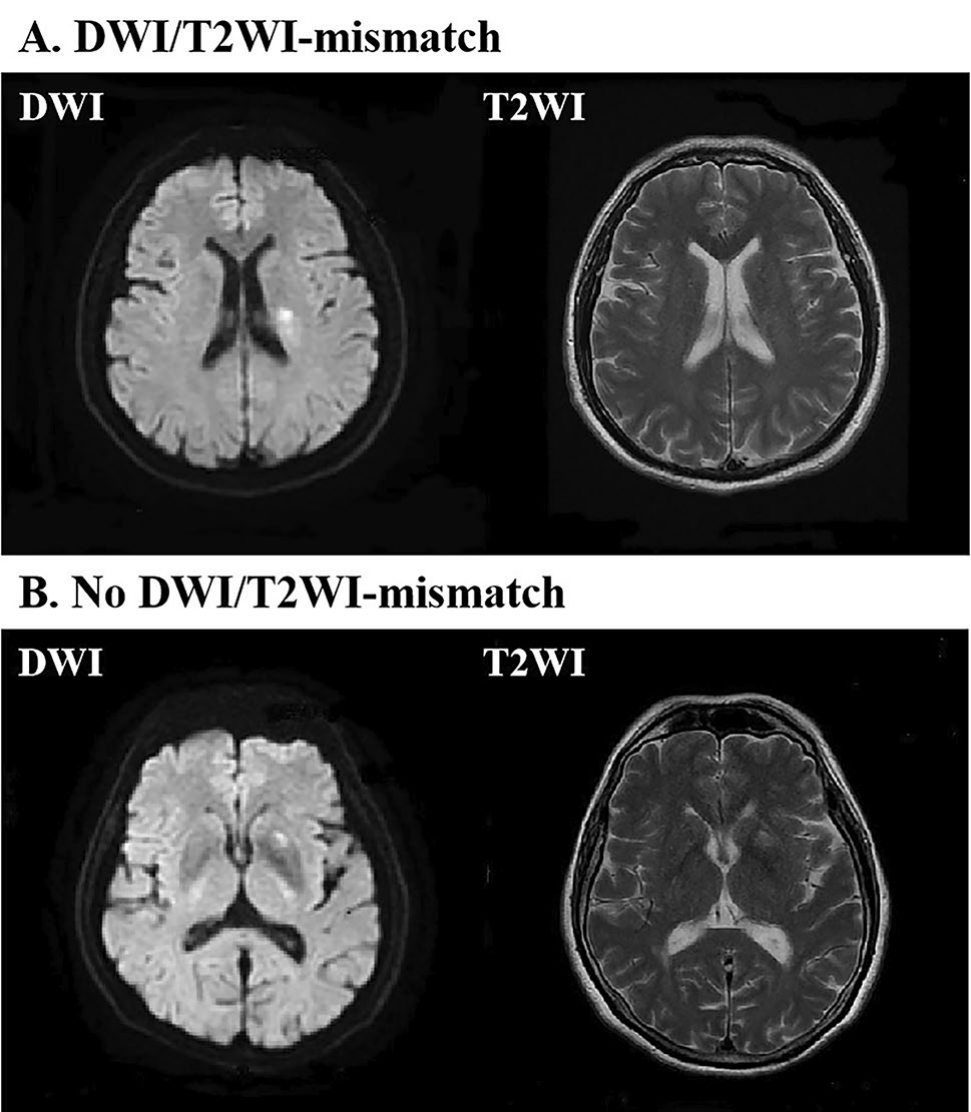
MRIs of patients with LSA stroke, with or without mismatch between DWI and T2WI within 12 h after onset of symptoms. (**A**) Pairs of images showing acute ischemic lesions on DWI but not on T2WI imaging (i.e., with DWI/T2WI-mismatch). (**B**) Pairs of images showing acute ischemic lesions on DWI together with a corresponding hyperintensity on T2WI imaging (without DWI/T2WI-mismatch).

Thus, the criteria for this study consisted of patients with LSA stroke who underwent thrombolysis by IV-TPA. The population comprised 104 (65%) patients treated within 4.5 h after the acute onset of symptoms, and 56 (35%) treated between 4.5 and 12 h. Neuroimaging evidence of LSA infarction within 12 h of acute stroke symptom onset was mandatory. The evidence consisted of a hyperintensity on diffusion-weighted sequences on MRI in the LSA paraventricular or basal ganglia region that corresponded to a clinical deficit (Fig. 1). Patients with any of the following were excluded from this study: imaging evidence of extracranial or intracranial carotid stenosis; proximal vessel occlusion; clot retrieval; or contraindications to IV-TPA.

The modified Rankin scale (mRS) was used to evaluate functional outcome at 90 days. A favorable clinical outcome was defined as mRS < 2 at 90 days. Symptomatic intracranial hemorrhage after intravenous thrombolysis was defined as any worsening of NIHSS (National Institutes of Health Stroke Scale) score ≥ 4 from intracerebral hemorrhage ^10^.

### Clinical and laboratory assessments

Noncontrast computed tomography and MRI (including DWI, T2WI, and magnetic resonance angiography [MRA]), which are the standard of practice in acute ischemia stroke intervention at our institution, were performed prior to thrombolysis and 24 h after TPA^10–13^. The MRIs were reviewed using standard PACS (Picture Archiving and Communications System) software. A negative/positive mismatch between the MRI DWI and T2WI was defined as an acute ischemic lesion on the DWI with/without a corresponding acute ischemic lesion on T2WI in the territory of the LSA (Fig. 1). The presence of a DWI/T2WI mismatch was independently assessed by a consultant neurologist and a senior stroke neurologist. A consensus was then reached. The fast MRI scan required 5–10 min to complete, and an additional 10 min were used to transfer or position the patient and to process the MRI. To administer the thrombolytic therapy as soon as possible, the necessary laboratory tests, informed consent, and drug preparation were completed concurrent with the fast MRI examination.

The recorded patient data were retrospectively analyzed and included the following: demographics; stroke onset time; risk factors; time from symptom onset to IV-TPA treatment; DWI and T2WI sequences; baseline NIHSS score; and mRS score at 90 days.

### MRI requirement

MRI was done simultaneously with the preparation of IV-TPA, and while awaiting lab results and the patient’s consent. GE 1.5 T HDXT Twinspeed was employed. Fast spin echo (FSE) was used for T2WI, with time repetition (TR) at 4000 ms, time of echo (TE) 102.0 ms/Ef, field of view (FOV) 24 cm × 18 cm, matrix 320 × 224, number of excitations 2 (NEX 2), thickness of 6 mm, and distance of each layer at 1 mm. The DWI parameters were: TR 6000 ms; TE 96 ms; thickness 6 mm; and b value at 0/1000 s/mm^2^. The parameters of 3-dimensional-time of flight-MRA (3D-TOF-MRA) were: TR 27 ms; flip angle 20°; thickness 1.4 mm; and bandwidth 25.

### Ethics statements

This study was previously approved by the Medical Ethics Committee of Tiantan Hospital, Capital Medical University, Beijing, China. For the years covered by this study, we conducted the enrollment with MR evaluation and obtained informed consent from all participants or their legally acceptable representative, and DWI/T2W mismatch was used as neuroimaging evidence for deciding whether to thrombolyse. All procedures involving human participants were conducted in accordance with the ethical standards of the institutional and/or national research committee and with the 1964 Helsinki Declaration and its later amendments or comparable ethical standards.

### Statistical analysis

Statistical analyses were performed using the Statistical Package for the Social Sciences, Version 24 (IBM, Armonk, New York). A *P*-value < 0.05 was considered statistically significant. Differences in patient characteristics among outcomes were tested by chi-squared test for categorical data, and the Mann–Whitney U test for continuous variables. Characteristics showing significant differences of *P* < 0.05 were subjected to binary logistic regression to assess an independent association with a 90-day outcome.

## Results

### General characteristics of patients by outcomes

The study population included 160 patients with LSA stroke who received treatment with IV-TPA within 12 h of symptom onset (Table 1). The median age was 59 years (26–80 years), 28.8% (46/160) were female, and the median baseline NIHSS score was 7 (interquartile range [IQR], 10–5). A favorable outcome (90-day mRS 0–1) was experienced by 73.1% (117/160) of the patients. Sixty-five percent (104/160) of the patients were treated within 4.5 h. No symptomatic bleeding complications were observed in any patient.

**Table 1:** Baseline and clinical characteristics of patients with LSA stroke by outcomes.

	Total	Favorable	Poor	*P*
Subjects, n	160	117	43	
Age, years^b^	59 (66–52)	59 (66–51)	59 (66–53)	0.89
Female^a^	46 (28.8)	34 (29.1)	12 (27.9)	0.89
Smoking^a^	72 (45)	52 (44.4)	20 (46.5)	0.82
Hypertension^a^	106 (66.3)	72 (61.5)	34 (79.1)	0.04
Diabetes^a^	21 (13.1)	15 (12.8)	6 (14.0)	0.85
Atrial fibrillation^a^	5 (3.1)	5 (4.3)	Nil	0.33
Previous stroke^a^	3 (1.9)	3 (2.6)	Nil	0.56
Onset-to-treatment time, min^b^	210 (303.8–152.5)	210 (302.5–151)	220 (315–160)	0.45
NIHSS score^b,c^	7 (10–5)	6 (8–5)	10 (14–7)	< 0.001
Admission glucose, mmol/L^b^	5.3(6.1–4.7)	5.2 (6.1–4.6)	5.3 (6.4–4.8)	0.32
SBP, mmHg^b^	158 (168–141.3)	153 (165.5–140)	160 (176–150)	0.002
DBP, mmHg^b^	90 (100–80)	90 (99–80)	96 (105–90)	0.005
IV-TPA 4.5–12 h	56 (35)	40(34.2)	16 (37.2)	0.72

^a^n (%).

^b^Median (IQR = Q3–Q1).

^c^Baseline.

Clinical characteristics in the overall population and when stratified by 90-day clinical outcome are listed in Table 1. Proportion of hypertension (61.5%, *P* = 0.04), baseline NIHSS score (median 6, *P* < 0.001), and admission systolic/diastolic blood pressure (S/DBP; median 153/90 mmHg, *P* = 0.002, 0.005) were significantly lower in patients with favorable outcome (mRS 0–1) compared with those with poor outcome (mRS 2–6) (79.1%, 10, 160/96 mmHg, respectively). No significant difference was seen in percent of patients treated at 4.5–12 h (*P* = 0.72) and onset to treatment time (OTT) (*P* = 0.45).

In patients with favorable outcomes, the proportion of history of diabetes was 8% (IV-TPA within 4.5 h) versus 22.5% (IV-TPA between 4.5 and 12 h; *P* = 0.02), which were the only significant differences between IV-TPA time. In patients with poor outcomes, no significant difference was seen between the IV-TPA time subgroups (Table 2).

**Table 2:** Baseline and clinical characteristics of patients stratified by outcomes and subdivided by onset-to-treatment.

	mRS (0–1)			mRS (2–4)		
	< 4.5 h	4.5–12 h	*P*	< 4.5 h	4.5–12 h	*P*
Subjects, n	77	40		27	16	
Age, years	59 (67–51.5)	59 (66–51.3)	0.90	58 (65–52)	60.5 (68.3–53.5)	0.42
Female	21 (27.3)	13 (32.5)	0.56	8 (32.1)	4 (25)	1.00
Smoking	36 (46.8)	16 (40)	0.49	14 (51.9)	6 (37.5)	0.36
Hypertension	44 (57.1)	28 (70)	0.18	22 (81.5)	12 (75)	0.91
Diabetes	6 (8)	9 (22.5)	0.02	5 (18.5)	1 (6.3)	0.51
Atrial fibrillation	3 (4)	2 (5)	1.00	Nil	Nil	–
Previous stroke	2 (2.7)	1 (2.5)	1.00	Nil	Nil	–
NIHSS score^a^	6 (9–5)	6 (7–5)	0.26	11 (16–8)	8 (13–6.3)	0.91
Glucose^b^, mmol/L	4.9 (6.1–4.5)	5.3 (6.3–4.9)	0.74	5.3 (6.5–4.8)	5.4 (6.3–4.8)	0.93
SBP, mmHg	154 (164.5–140)	151.5 (169–138.5)	0.92	163 (180–147)	160 (169–150)	0.46
DBP, mmHg	90 (100–80)	90 (97.5–81.3)	0.45	100 (106–90)	90 (100–88.5)	0.14

Reported as n (%) or median (IQR = Q3–Q1), or as indicated.

^a^Baseline.

^b^At admission.

Patient’s clinical characteristics were stratified according to whether IV-TPA was administered within 4.5 h, or 4.5–12 h of symptom onset (Table 3). No significant differences were noted in the clinical characteristics by these stratifications.

**Table 3:** Clinical characteristics and outcomes by onset-to-treatment time.

	< 4.5 h	4.5–12 h	*P*
Subjects, n	104	56	
Age, years	59 (66.7–52)	59 (66–52)	0.75
Female	29 (27.9)	17 (30.4)	0.74
Smoking	49 (47.1)	20 (35.7)	0.29
Hypertension	66 (63.5)	40 (71.4)	0.31
Diabetes	11 (10.6)	8 (14.3)	0.19
Atrial fibrillation	3 (2.9)	2 (3.6)	1.00
Previous stroke	2 (1.9)	1 (1.8)	1.00
NIHSS score^a^	7 (11–5)	7 (8–5)	0.13
Glucose^b^, mmol/L	5.1 (6.1–4.6)	5.3 (6.3–4.9)	0.15
SBP, mmHg	160 (168–140.3)	156 (169–143.3)	0.89
DBP, mmHg	90 (100–80)	90 (98.5–85.3)	0.96
**mRS**^**c**^			
0	59 (56.7)	25 (44.6)	0.14
1	18 (17.3)	15 (26.8)	0.16
2	14 (13.5)	12 (21.4)	0.19
3	13 (12.5)	3 (5.4)	0.25
4	0	1 (1.8)	0.35

Reported as n (%) or median (IQR = Q3–Q1), or as indicated.

^a^Baseline.

^b^At admission.

^c^No patient had a mRS > 4.

Clinical characteristics and outcomes stratified by a 4.5-h time window of IV-TPA and subdivided by mRS outcomes are shown in Table 4. In patients given IV-TPA within 4.5 h, proportion of hypertension (57.1%, *P* = 0.04), NIHSS score (median 6, *P* < 0.001) and admission systolic/diastolic blood pressure (median 154/90 mmHg, *P* = 0.04) remained lower in the mRS 0–1 group compared with the mRS 2–6 (81.5%, 11 and median 163/100 mmHg, respectively). In patients given IV-TPA between 4.5 to 12 h, the median NIHSS score was 6 (mRS 0–1) compared with 8 (mRS 2–6; *P* = 0.004), which was the only significant difference between outcomes (Table 4).

**Table 4:** Baseline and clinical characteristics of patients with LSA stroke between IV-TPA within 4.5-h and 4.5–12 h and subdivided by outcomes.

	< 4.5 h			4.5–12 h		
	mRS (0–1)	mRS (2–4)	*P*	mRS (0–1)	mRS (2–4)	*P*
Subjects, n	77	27		40	16	
Age, years	59 (67–51.5)	58 (65–52)	0.73	59 (66–51.3)	60.5 (68.3–53.5)	0.51
Female	21 (27.3)	8 (32.1)	0.81	13 (32.5)	4 (25)	0.82
Smoking	36 (46.8)	14 (51.9)	0.65	16 (40)	6 (37.5)	0.86
Hypertension	44 (57.1)	22 (81.5)	0.04	28 (70)	12 (75)	0.96
Diabetes	6 (7.8)	5 (18.5)	0.23	9 (22.5)	1 (6.3)	0.30
Atrial fibrillation	3 (3.9)	Nil	0.57	2 (5)	Nil	1.00
Previous stroke	2 (2.6)	Nil	1.00	1 (2.5)	Nil	1.00
Onset-to-treatment time, min	170 (210–130)	185 (215–135)	0.52	330 (399–300)	352.5 (463.8–292.5)	0.56
NIHSS score^a^	6 (9–5)	11 (16–8)	< 0.001	6 (7–5)	8 (13–6.3)	0.004
Glucose^b^, mmol/L	4.9 (6.1–4.5)	5.3 (6.5–4.8)	0.15	5.3 (6.3–4.9)	5.4 (6.3–4.8)	0.78
SBP, mmHg	154 (164.5–140)	163 (180–147)	0.005	151.5 (169–138.5)	160 (169–150)	0.16
DBP, mmHg	90 (100–80)	100 (106–90)	0.003	90 (97.5–81.3)	90 (100–88.5)	0.61

Reported as n (%) or median (IQR = Q3–Q1), or as indicated.

^a^Baseline.

^b^At admission.

### Predictors of favorable outcome (Table 5)

**Table 5:** Predictors of favorable outcome.

	OR (95% CI)	*P*
**In total patients (including OTT, history of hypertension, NIHSS, SBP and DBP)**		
NIHSS score^a^	1.348 (1.196–1.520)	< 0.001
SBP^b^	1.042 (1.018–1.066)	< 0.001
**In patients iv-tPA within 4.5 h (including OTT, history of hypertension, NIHSS, SBP and DBP)**		
NIHSS score^a^	1.373 (1.185–1.592)	< 0.001
SBP^b^	1.053 (1.022–1.084)	0.001
**In patients iv-tPA between 4.5 and 12 h (including OTT and NIHSS)**		
NIHSS score^a^	1.385 (1.108–1.731)	0.004

^a^Baseline.

^b^At admission.

The logistics analysis showed that lower admission SBP and NIHSS score were independently associated with a favorable outcome in patients overall (admission SBP: odds ratio [OR] 1.042, 95% CI 1.018–1.066, *P* < 0.001; baseline NIHSS score: OR 1.348, 95% CI 1.196–1.520, *P* < 0.001). However, history of hypertension was not a significant predictive factor.

Patient’s clinical characteristics were stratified according to whether IV-TPA was administered within 4.5 h, or 4.5 to 12 h of symptom onset and subdivided by outcomes. In patients given IV-TPA within 4.5 h, admission SBP and NIHSS score were negatively associated with favorable outcome, with adjusted ORs of 1.053 (95% CI 1.022–1.084, *P* = 0.001) and 1.373 (95% CI 1.185–1.592, *P* < 0.001), respectively. In patients given IV-TPA between 4.5 and 12 h, the NIHSS score was an independent predictor of 90-day mRS < 2 with an adjusted OR of 1.385 (95% CI 1.108–1.731, *P* = 0.004).

No difference in OTT was found between patients who had favorable outcome and poor outcome, or patients who received IV-TPA < 4.5 h and 4.5–12 h and subdivided by favorable outcome and poor outcome, as well as a logistic regression respectively. This further strengthens the concept that in MRI-based treatment, the traditional dependency of outcome on time gets loosened.

## Discussion

This retrospective analysis determined that the outcomes of MRI-guided selection of IV thrombolysis for treatment of patients with LSA stroke were similar, whether conducted within 4.5 h, or 4.5 up to 12 h, after symptom onset. Lower admission SBP and NIHSS score are two protective factors for patients to reach a good functional outcome. Lower admission SBP and NIHSS score were, for the overall cohort, predictive factors to reach good functional outcome, however the association between SBP and favorable outcome was more evident in the early time window.

LSAs are perforating vessels originating from the middle cerebral artery. They are some of the most vital vascular structures, are terminal, and the sites of cerebral vascular disease^14^. Hypertension can alter the vascular structure, mechanics, and function of small arteries and arterioles^15,16^. These changes disturb the structure and function of microcirculation in the LSAs, and are associated with increased vascular resistance and reduced blood flow through the microvessels^17^. Cumulative microvascular damage from hypertension has a deleterious effect on the number of LSA stems^18^, endothelial cell senescence, collateral rarefaction^19^, and cerebral blood flow, and accelerates the neurological deficits of LSA stroke.

For acute LSA stroke, when blood pressure increases beyond the specific range of cerebral autoregulation, constant cerebral blood flow is impaired and secondary injury occurs^20^. Of the baseline biological and anamnestic parameters investigated in this study, SBP at admission affected outcome the most, especially in the early time window.

High admission SBP favors an acute hypertensive response of the ischemic area. Changes in pressure pulsations lead to a fast and dynamic response, resulting in progressive vasoconstriction of arterioles before BP exceeds the upper limit of autoregulation. After that, vasodilation is formed which causes an increase of cerebral blood flow, resulting in the dysfunction of blood–brain barrier, cerebral edema, and exacerbation of cerebral infarction^21^. In addition, hypoperfusion correlates with high SBP. The cortical and hippocampal cerebral blood flow declined with increasing SBP in an entire study group (with and without hypertension) across the entire blood pressure spectrum^22^. These factors likely predispose patients with higher baseline blood pressure, treated with intravenous thrombolysis, to poor functional outcomes.

The present findings are in accord with those of the European Cooperative Acute Stroke Study ECASS-II^23^, which documented poor outcomes in patients with acute ischemic stroke and elevated baseline SBP levels. A lack of hypertensive history, or lower SBP on admission, may be protective in cases of brain ischemia, due to greater arterial compliance, normalized cerebrovascular autoregulation, less decrease in cerebral blood flow during ischemia at the periphery of the lesion, and enhancement of collateral circulation during hypoperfusion.

Few previous studies have focused on the validity of IV-TPA in LSA stroke, because of the unpopularity of using magnetic resonance during the emergency. Thus, at admission acute LSA stroke may be clinically diagnosed but rarely confirmed by magnetic resonance. The WAKE-UP trial provided evidence of the benefit of IV-TPA for patients with acute stroke, unknown time of symptom onset, and a mismatch between MRI findings on DWI and FLAIR^9^. The results of a secondary post hoc analysis showed that IV-TPA was safe, and improved the functional outcome of patients with lacunar infarct; the outcome was similar for patients with other stroke subtypes^24^.

In the present study, LSA stroke was identified in patients using emergency magnetic resonance. The safety and effectiveness outcomes of IV-TPA for those treated from 4.5 to 12 h after the onset of symptoms were comparable to that of patients treated within 4.5 h. In this study, T2WI was used rather than FLAIR, because performing T2W images (requiring 50 s) is ~ 100 s faster than FLAIR^10^. Although cytotoxic edema on T2WI is less clearly shown because of the greater artefact and partial volume effect from cerebral spinal fluid^25^, LSA infarctions are distant enough from cerebral spinal fluid and may be weakly influenced by those limitations. In addition, the faster image formation of T2WI can save onset-to-treatment time and reduce the influence of motion in less cooperative patients during the magnetic resonance examination.

The results of this study are limited by the retrospective nature of the analysis and the small number of patients from a single center. Whether IV-TPA between 4.5 and 12 h is superior to that of other therapies was not analyzed, because randomization for different treatments was not possible. No deaths or symptomatic intracerebral hemorrhages were recorded in patients treated either within the 4.5-h time window or patients treated beyond this time window. However, definite statements regarding the safety of MRI-guided TPA in LSA stroke in the extended time window cannot be made based on this study and further investigation in warranted in a larger cohort.

In conclusion, the outcomes of intravenous TPA for patients who were selected based on DWI/T2WI mismatch and treated 4.5–12 h after symptom onset were comparable to that of patients treated within 4.5 h. In addition, lower admission NIHSS scores and lower admission systolic blood pressure were independent predictors of good functional outcome for the entire cohort. Our findings also suggest that patients who undergo MRI-guided thrombolysis within 12 h of isolated lenticulostriate artery stroke will experience better outcomes if there is low admission systolic blood pressure and/or low baseline NIHSS score.
